# One- vs. Two-Stage Revision for Periprosthetic Shoulder Infections: A Systematic Review and Meta-Analysis

**DOI:** 10.3390/antibiotics13050440

**Published:** 2024-05-14

**Authors:** Mohamad Bdeir, Aimée Lerchl, Svetlana Hetjens, Andreas Schilder, Sascha Gravius, Tobias Baumgärtner, Ali Darwich

**Affiliations:** 1 Department of Orthopaedic and Trauma Surgery, University Medical Centre Mannheim, Medical Faculty Mannheim, University of Heidelberg, Theodor-Kutzer-Ufer 1–3, 68167 Mannheim, Germany; aimee.lerchl@hotmail.com (A.L.); andreas.schilder@medma.uni-heidelberg.de (A.S.); sascha.gravius@umm.de (S.G.); tobias.baumgaertner@umm.de (T.B.); alidarwich@mail.com (A.D.); 2 Institute of Medical Statistics and Biomathematics, University Medical Centre Mannheim, Medical Faculty Mannheim, University of Heidelberg, Theodor-Kutzer-Ufer 1–3, 68167 Mannheim, Germany; svetlana.hetjens@medma.uni-heidelberg.de

**Keywords:** periprosthetic shoulder infection, revision, one stage, two stage, shoulder arthroplasty, failure

## Abstract

Periprosthetic shoulder infection (PSI) remains a challenging complication after shoulder arthroplasty. Therapeutic options include one- or two-stage revision, irrigation and debridement, and resection arthroplasty. With our systematic review and meta-analysis, we aimed to compare one- and two-stage revisions for periprosthetic shoulder joint infections and determine the most appropriate therapeutic procedure. We performed an extensive literature search in PubMed, Ovid Medline, Cochrane Library, Web of Science, and CINAHL and filtered out all relevant studies. The meta-analysis was performed using the random-effects model, heterogeneity was analyzed using I^2^, and publication bias was assessed using the Egger’s test. A total of 8 studies with one-stage revisions, 36 studies with two-stage revisions, and 12 studies with both one-stage and two-stage revisions were included. According to the random-effects model, the reinfection rate for the entirety of the studies was 12.3% (95% Cl: 9.6–15.3), with a low-to-moderate heterogeneity of I^2^ = 47.72%. The reinfection rate of the one-stage revisions was 10.9%, which was significantly lower than the reinfection rate of the two-stage revisions, which was 12.93% (*p* = 0.0062). The one-stage revision rate was significantly lower with 1.16 vs. 2.25 revisions in the two-stage revision group (*p* < 0.0001). The postoperative functional outcome in one-stage-revised patients was comparable but not statistically significant (*p* = 0.1523). In one- and two-stage revisions, most infections were caused by *Cutibacterium acnes*. In summary, our systematic review and meta-analysis show the superiority of single-stage revision regarding reinfection and revision rates in periprosthetic shoulder joint infection.

## 1. Introduction

Periprosthetic shoulder joint infection (PSI) is a devastating complication after joint arthroplasty and is associated with significant morbidity [1]. PSI is a common cause of surgical revision and persistent shoulder pain [2]. It constitutes a great burden to the health care system and is also associated with unsatisfactory functional outcomes and impairment [3]. After primary arthroplasty of the shoulder, the incidence of PSI ranges from 1% to 4% [4,5]. After revision arthroplasty of the shoulder joint, the incidence increases from 4% to 15% [4,5]. In addition, mortality rates of up to 3% have been observed within 90 days after revision shoulder arthroplasty in older patients [6]. Typical causative pathogens for PSI include coagulase-negative staphylococci (CNS)*, Cutibacterium acnes* (*C. acnes*), *Staphylococcus aureus*, and *Staphylococcus epidermidis* [4,7]. Various comorbidities such as obesity, diabetes mellitus, rheumatic diseases, iron-deficiency anemia, and previous injections with corticosteroids can increase the risk of PSI [8,9,10,11].

A timely, reliable diagnosis (e.g., by intraoperative smears) and optimal therapy are major challenges of PSI [5]. Based on the Musculoskeletal Infection Society criteria, a PSI exists when a sinus tract is associated with the prosthesis, or a pathogen is isolated by culture from at least two separate tissue or fluid samples obtained from the affected prosthetic joint [12]. Although the recently defined criteria of the International Consensus Meeting (ICM) on orthopedic infections allow a classification into definite, probable, possible, and improbable infections, many of the cited publications are nevertheless based on specifically defined and variable criteria of the respective authors [3,13]. The paucity of established therapeutical algorithms for PSI in the literature represents a challenge for surgeons and a major limitation in treatment [14]. With regard to the management of PSI, surgical therapy is based on the guidelines for PJI of the knee or hip, although the spectrum of infectious microorganisms of PJI varies between the shoulder and knee/hip, and there are also considerable anatomical and biomechanical differences [15]. The therapy of PSI is based on the therapeutical guidelines of periprosthetic hip and knee infections [16]. Possible treatment options for PSI include preservation of the implant after extensive irrigation and debridement, one- as well as two-stage replacement of the joint prosthesis, and resection arthroplasty [13]. A two-stage replacement includes removal of the infected implant with subsequent irrigation and debridement, the insertion of an antibiotic spacer, and delayed prosthesis replacement [5,16].

An important advantage of one-stage revision is the reduced damage to soft tissue, which is thought to lead to better outcomes and lower reinfection rates. Furthermore, one-stage revisions are associated with shorter duration of antibiotic therapy and shorter hospital stays with lower treatment costs [17]. Severely ill patients with a high surgical risk also benefit from the one-stage procedure [18]. In patients with glenoid bone defects, a one-stage revision is inferior to a two-stage revision [19]. In such cases, bone grafting and glenoid defect reconstruction is usually performed first, and the glenoid component is inserted in a second procedure after the graft has healed in order to achieve a stable reconstruction of the glenoid [20]. Regarding the two-stage revision, soft tissue damage is the most important disadvantage. Further drawbacks include longer duration of antimicrobial treatment, higher number of surgical revisions, and longer hospital stays as well as higher rates of postoperative complications [15,17,18]. On the other hand, two-stage revision is thought to be associated with higher infection resolution rates and lower infection recurrence rates as well as better functional outcomes [18,21].

However, the evidence to date of one- or two-stage prosthesis revision in PSI is inconclusive, and the experience is still inferior in comparison to experience in the treatment of periprosthetic infections in other joints [7]. The purpose of this systematic review is to compare one-stage and two-stage revisions in PSI regarding the causative pathogen, functional outcome, and rate of reinfection. The main outcome parameter of this review was the evaluation of reinfection rates and secondary outcomes included revision rates and functional outcome.

## 2. Results

The current meta-analysis includes 8 studies investigating only single-stage revisions [21,22,23,24,25,26,27,28], 36 studies investigating only two-stage revisions [4,29,30,31,32,33,34,35,36,37,38], and 12 studies investigating both one- and two-stage revisions [8,16,19,39,40,41,42,43,44,45,46,47] (Table 1).

**Figure 1:**
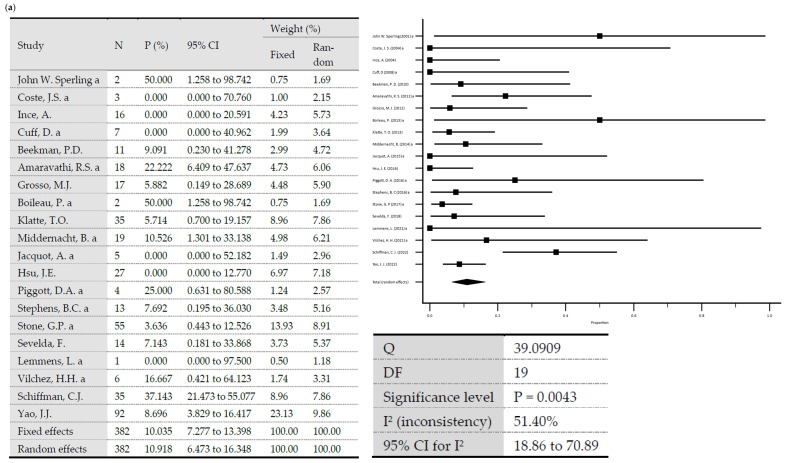

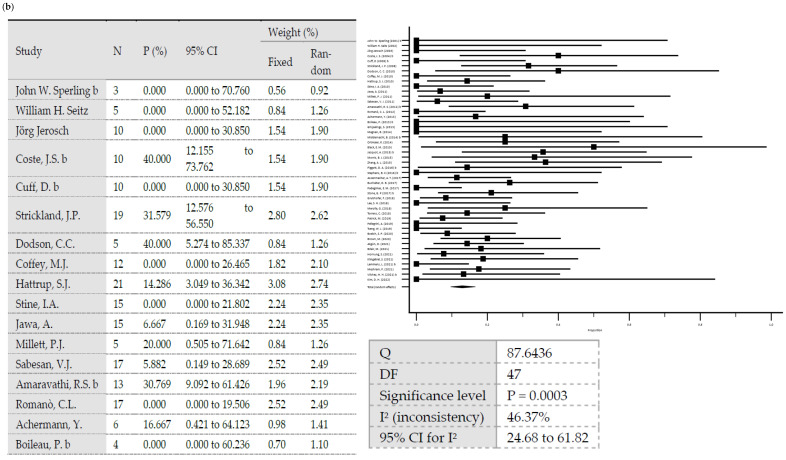

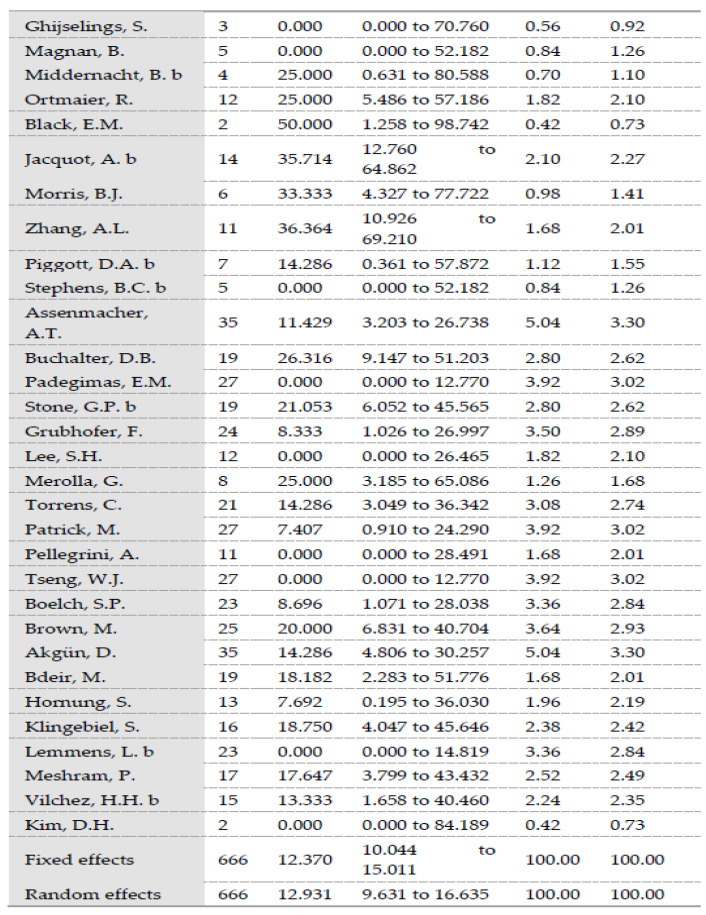
(**a**) Forest plots representing reinfection rates after one-stage revisions. (**b**) Forest plots representing reinfection rates after two-stage revisions (N, sample size; P, % proportion; CI, confidence interval; W, % weight).

A total reinfection rate of 12.3% was observed (95% Cl = 9.6–15.3). In one-stage revisions, reinfection rates of 10.9% (95% Cl = 6.47–16.35) were observed versus 12.93% (95% Cl = 9.63–16.63) after two-stage revisions. The difference was statistically significant (*p* = 0.0062). Heterogeneity testing of all included studies showed low heterogeneity, with I^2^ values of 47.72% (95% Cl: 30.55–60.64) (*p* < 0.0001) [70]. Testing of the group of studies included in one-stage revisions and those included in two-stage revisions also showed low heterogeneity, with I^2^ values of 51.4% (95% Cl: 18.86–70.89) (*p* = 0.0043) and 46.37% (95% Cl = 24.68–61.82) (*p* = 0.0003), respectively. Egger’s test showed no significance in evaluating publication bias considering all included studies (*p* = 0.076) and considering the included studies in one-stage revisions (*p* = 0.3157) and in two-stage revisions (*p* = 0.23) (Figure 1).

Regarding revision rates, the one-stage revision group showed significantly lower rates with 1.16 ± 0.18 revisions versus 2.25 ± 0.33 revisions in the two-stage revision group (*p* < 0.0001). The evaluation of the microbiological data shows a predominance of *Cutibacterium acnes* in both one- and two-stage revisions. In one-stage revisions, *Staphylococcus aureus* is the second most frequent pathogen, followed by *Staphylococcus epidermidis*, coagulase-negative staphylococci (CNS), *Mycobacterium tuberculosis*, *Staphylococcus capitis*, MRSA, and various streptococci and enterococci species. In two-stage revisions, *Staphylococcus epidermidis* is the second most common pathogen, followed by CNS then *Staphylococcus aureus*, MSSA, *Pseudomonas aeruginosa*, MRSA, and various enterococci and streptococci species (Table 2).

The mean age of all patients included in the meta-analysis was 65.76 ± 4.28 years (range 54–71.5). The mean follow-up was 44.91 ± 31.79 months for one-stage revisions and 38.74 ± 16.91 months for two-stage revisions. The age of the patients as well as follow-up periods did not significantly differ between both groups (*p* = 0.7896 and *p* = 0.4425).

The C-reactive protein (CRP) value was reported in only some of the included studies (32/56). For one-stage revisions, the mean value was 3.7 ± 4.12 mg/dL. For two-stage revisions, the mean value was 3.85 ± 3.07 mg/dL, without significant difference (*p* = 0.9191).

Similarly, functional and clinical scores were reported in only some of the included studies (50/56). The constant score and the postoperative abduction were the most frequently used parameters. A constant score of 51.82 ± 9.17 points was observed after one-stage revisions and 45.22 ± 12.07 points after two-stage revisions. The differences were not significant (*p* = 0.1523). Similarly, the mean postoperative abduction and elevation of the shoulder joint were 103.38 ± 40.31° and 101.47 ± 20.01°, respectively, after one-stage revisions and 87.22 ± 20.61° and 93.42 ± 17.20°, respectively, after two-stage revisions, without reaching statistical significance (*p* = 0.4208 and *p* = 0.5530, respectively).

The American Shoulder and Elbow Surgeons Shoulder Score (ASES) and simple shoulder test were reported in some of the included studies. The ASES was reported with 57.30 ± 3.72 points in the one-stage revision group versus 67.06 ± 5.85 points in the two-stage revision group. On the other hand, the simple shoulder test (SST) score was reported with 5.34 ± 1.72 points after one-stage revisions versus 11.43 ± 18.50 points after two-stage revisions. The differences shown in both scores did not reach statistical significance (*p* = 0.0326 and *p* = 0.3526, respectively). The data of the outcome is listed in Table 3.

## 3. Discussion

Due to the continuous increase in the number of patients with shoulder joint prosthesis, the question of the best possible therapy for periprosthetic shoulder joint infection (PSI) is becoming increasingly important [71]. In addition to irrigation and debridement, one- or two-stage revision or, alternatively, resection arthroplasty are possible therapeutic options [72]. The question of whether one- or two-stage revision should be considered the preferred procedure is answered differently in the literature [67,72]. The aim of this meta-analysis was to investigate the outcomes of one-stage vs. two-stage revision in PSI and highlight complications to better answer this question and optimize the therapy of PSI.

The overall reinfection rate of 12.3%, which is independent of the revision procedure, is slightly higher than the reinfection rate of 8.9% determined by Belay et al. in a comparable systematic review [73]. The systematic review by Belay et al. excluded studies with less than 2 years of follow-up. Our systematic review and meta-analysis included studies from 12 months follow-up. This could have led to the differences in the reported reinfection rates. Furthermore, the difference in reinfection rates could be due to a greater number of studies reporting two-stage revision, significant heterogeneity across subgroups, and a likely reporting bias favoring the reporting of smaller infection rates [74]. In addition, 12-month infection eradication success rates are reported in the majority of current studies. This could also lead to the incorrectly reported low reinfection rates. Future studies could be designed to compare the long-term success of one- or two-stage revision for shoulder PJI in terms of infection eradication and alternative techniques for measuring overall infection eradication to achieve a better clinical outcome for patients.

The comparison of reinfection rates after one- or two-stage revision showed a significant advantage of one-stage revision (*p* = 0.0062). Comparable results can be found in the literature, although the advantage of one-stage revision is mostly reported as not significant [72,73]. Also, in our meta-analysis, the revision rates in the one-stage revision group were significantly lower, with 1.16 ± 0.18 revisions versus 2.25 ± 0.33 revisions in the two-stage revision group (*p* < 0.0001). This is in accordance with the studies in the literature [43,72,73]. Nevertheless, there are several factors that may influence the result. The one-stage revision has several benefits for the patient. A big advantage is that this treatment results in less soft tissue damage and therefore fewer surgical complications than with two-stage revision [19,75]. The lower reinfection rate during one-stage revision results in better clinical functional results. Furthermore, it is generally associated with lower treatment costs, shorter hospital stays, and shorter systemic antibiotic therapy. This approach has less soft tissue damage and less surgical comorbidity. All of these factors have a positive effect on the satisfaction and psyche of the patient, which plays a major role in the success of the therapy [17].

The one-stage revised patients in our meta-analysis had a lower CRP value of 3.7 mg/dL compared to the two-stage revised patients with 3.85 mg/dL, without a significant difference. This could indicate a less severe infection with a more promising successful therapy in patients with one-stage revision. This assumption matches the recommendation in the literature to perform one-stage revisions only in case of a known and low-virulent pathogen [76]. Furthermore, it should be noted that the number of studies and patients with one- or two-stage revision is not identical. The study design and patient population also vary between the different studies. Thus, the variability between studies limits the direct comparability of our results.

In the current meta-analysis, *C. acnes* was shown to be the most common PSI-causing microorganism in both one-sage and two-stage revisions, followed by *Staphylococcus aureus*, *Staphylococcus epidermidis*, and CNS in one-stage revisions and by *Staphylococcus epidermidis* and CNS in two-stage revisions. This is in line with data in the literature showing the predominance of *C. acnes* as the main pathogen causing PSI [15,31,73]. The proportion of MRSA as causing agent is, however, lower than that reported in the literature [73]. This may be caused by the fact that some studies [31,36] only included *C. acnes* infections, which may have led to an overestimation of the proportion of PSI caused by *C. acnes* and to an underestimation of the numbers of the remaining causative pathogens.

The average value shows a postoperative abduction of 103.38° after one-stage revision and is similar to that described in the literature [22,72]. Lemmens et al. reported values of 120° abduction after one-stage revision regime of PSI in 42 patients [19]. Postoperative abduction after two-stage revision is comparable to data in the literature, with a mean value of 87.22° [19]. In our meta-analysis, the difference in abduction was not significant. The deviation of our results on postoperative abduction after single-stage revision from the data in the literature could be due to the variability of the prostheses implanted. For example, the study by Ince et al. shows less restriction of abduction ability after inverse prosthesis compared with abduction ability after hemi-endoprosthesis [22]. Only few of the included studies reported data on the design and type of the implanted prosthesis; for this reason, a sub-analysis in this regard was not performed, and the confirmation of the correlation between clinical outcome and prosthesis type was not possible.

The constant score shows a mean value of 51.82 points for the one-stage and 45.22 points for the two-stage revision. The differences were not statistically significant; however, the results were similar to those reported in the literature [19,73].

These arguments represent the weaknesses of our systematic review and meta-analysis, which must be considered when interpreting the results. Nevertheless, our results clearly show that one-stage revision is an efficient therapeutic procedure for the treatment of PSI and is not inferior to two-stage revision. One-stage revision is also more cost-effective and avoids additional surgery, with its accompanying risks and complications [22].

Some confounding factors may have affected the results of this meta-analysis, for example, the heterogeneity of the parameters analyzed and the data collected in the included studies. Not all studies reported the same parameters to the same extent. Also, the follow-up time did indeed not significantly differ between the studied groups but varied from 12 to 164.5 months. Furthermore, the studies were carried out over a period of approximately 20 years with the respective different therapeutic approaches and convictions at that time. However, given the paucity of data, the inclusion of only recent studies or only studies with a high number of patients would have negatively affected the statistical significance and informative value of the meta-analysis.

A reasonable total number of patients were analyzed in this meta-analysis. Nevertheless, some studies included only small numbers of patients. This may have acted as a confounding factor.

In addition, it must be taken into consideration that various factors such as the time of infection, the causative pathogen, the severity of infection, and the patient’s comorbidity influence the choice of therapeutic procedure. Since these influencing factors also played a role in the choice of therapeutic procedure in the studies we included, a resulting bias cannot be ruled out. A potential additional cause for bias is the fact that the number of one-stage revision studies is smaller than the number of two-stage revision studies, which affects the conclusion. Another risk of bias is the dependence of the results on the center where the therapy was carried out.

As a further limitation of this review, it was not possible to distinguish between usually simple cases treated in one-stage revision and patients with previous revisions or difficult-to-treat pathogens cases treated in two-stage revision. Such details about the included patients were not provided. Additionally, none of the included studies mentioned that the choice of surgical treatment was based on these factors. Such an algorithm is definitely a selection bias and must be taken into consideration in the evaluation of the end outcome.

## 4. Materials and Methods

This systematic review is based on the PRISMA (Preferred Reporting Items for Systematic reviews and Meta-Analyses) guidelines and checklists [70].

### 4.1. Search Strategy

The systematic literature search was conducted by a qualified medical librarian and was performed in the following databases: PubMed, Ovid Medline, Cochrane Library, Web of Science, and CINAHL.

The following key terms were included in the search:

“Shoulder” AND “Astroplasty” or “total joint” or “replacement” or “prosthesis” or “periprosthetic” AND “Prosthesis-Related Infections” or “Infection” or “Reinfection” or “positive culture” AND “1-stage” or “2-stage” or “one-stage” or “two-stage” or “single stage” or “Resection” or “Exchange” or “Explantation” or “re-implantation” or “reimplantation” or “spacer” or “Reoperation” or “revision” or “failure“ or “outcome”.

### 4.2. Study Selection and Eligibility Criteria

Using this search strategy, 1316 studies were identified. From the total number of these studies, duplicates were excluded first. The title and abstract of the remaining studies were assessed by two of the authors independently (M.B. and T.B.), with respect to the previously defined exclusion criteria. In a next step, the full texts of the remaining studies were read and checked independently by two of the authors (M.B. and A.D.)for their suitability for the systematic review. Studies were included in which only one- or two-stage surgical revision was presented as a treatment for PSI after shoulder arthroplasty. Furthermore, only studies that investigated the reinfection rate in these patients and other clinical outcomes were included. A follow-up of at least 12 months was a criterium for inclusion in the review. All studies in non-English language; case reports (65); reviews (185); studies with content related to other joints such as hip, knee, wrist, finger joints, or elbow (349); studies with animal experiments; studies with a follow-up of less than 12 months; and studies with a treatment approach other than one- or two-stage prosthesis replacement were excluded.

**Figure 2:**
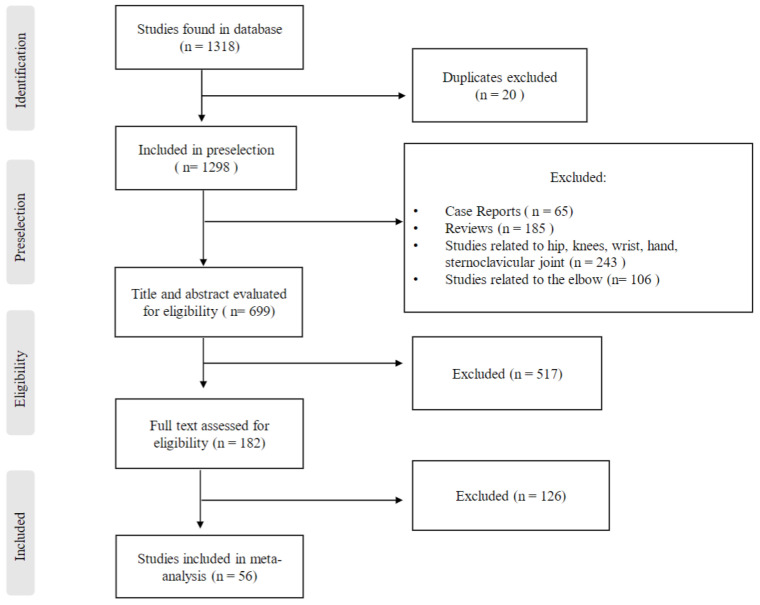
Study selection and eligibility criteria.

This exclusion process resulted in a selection of 56 studies that formed the basis of this systematic review (Figure 2).

### 4.3. Statistical Analysis

Data that were useable for pooled analysis due to their comparability (e.g., the revision procedure data) were included in the meta-analytic calculations. Continuous data that were not useable for pooled analysis were analyzed by inverse-variance model and reported as mean values. Statistical analysis was performed by a qualified statistician with special expertise in meta-analysis.

**Figure 3:**
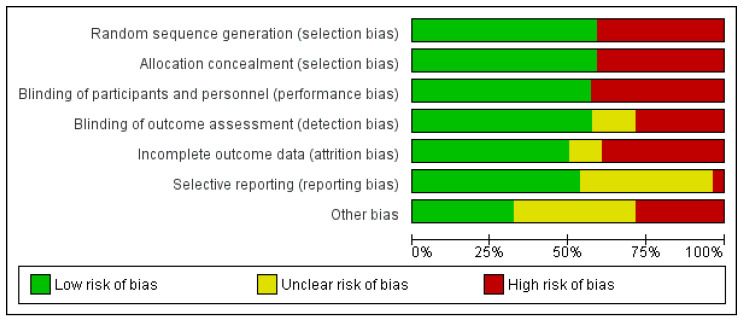
Methodological quality of the studies included in the meta-analysis.

The studies included in the meta-analysis were analyzed for heterogeneity and publication bias using the statistical software MedCalc (MedCalc^®^ Statistical Software version 20.111 (MedCalc Software Ltd., Ostend, Belgium; https://www.medcalc.org; accessed on 9 October 2022). The heterogeneity of the results was tested by the I^2^ index, where, according to Higgins et al., a value of more than 25% to 50% is classified as low, from more than 50% to 75% as moderate, and from more than 75% as high [77]. Heterogeneity was taken into consideration by using the random-effects model. Publication bias was determined using Egger’s test and reported as significance level (Figure 3). Continuous data were reported, according to Hozo et al., as mean values and standard deviation [78]. MedCalc statistical software was used for meta-analysis calculations, and SAS software (version 9.4 (SAS Institute INC., Cary, NC, USA)) was used for mean and standard deviation data. For the SAS software calculations, the number of patients (n) was used for weighting. A *p*-value lower than 0.05 was considered statistically significant.

## 5. Conclusions

The present meta-analysis shows that one-stage revision of PSI has a lower reinfection and revision rates compared to two-stage revisions. However, these results should be interpreted cautiously, especially regarding selection bias. A biased selection of the treatment algorithm such as one-stage revision in simple cases and two-stage revision in complex cases cannot be completely ruled out. Our systematic review and meta-analysis should be used as a basis for future studies in which the results can be confirmed by a controlled–randomized study design.

## Figures and Tables

**Table 1:** Overview of the basic data of the included studies.

Study	Year	No. of Patients	Mean Age (Years)	Follow-Up (Months)	One-Stage: a Two-Stage: b	Antibiotic-Impregnated Cement Spacer	
John W. Sperling [8]	2001	2	54	24	a	-	-
		3			b	No	-
William H. Seitz [29]	2002	5	62	49.5	b	Yes	Tobramycin (2 g)
Jörg Jerosch [30]	2003	10	71	18	b	Yes	NR
Coste, J.S. [16]	2004	3	64	34	a	-	-
		10			b	Yes	NR
Ince, A. [22]	2004	16	68	69.6	a	-	-
Cuff, D [39]	2008	7	67	43	a	-	-
		10			b	No	-
Strickland, J.P. [31]	2008	19	62	35	b	No	-
Beekman, P.D. [23]	2010	11	62	24	a	-	-
Dodson, C.C. [4]	2010	5	60.1	48	b	Yes	NR
Coffey, M.J. [32]	2010	12	58.9	18.3	b	Yes	Gentamicin/Vancomycin
Hattrup, S.J. [33]	2010	21	66.9	49.2	b	Yes	Gentamicin (4.8 g)/Vancomycin (2 g)/Cefazolin (2 g)
Stine, I.A. [34]	2010	15	61	24	b	Yes	Tobramycin (1.2 g)/ Vancomycin (1 g)
Jawa, A. [35]	2011	15	63	27.6	b	Yes	Tobramycin (3 g)/ Gentamicin (2 g)
Millett, P.J. [36]	2011	5	57.8	20.4	b	Yes	NR
Sabesan, V.J. [37]	2011	17	67.6	46.2	b	Yes	NR
Amaravathi, R.S. [40]	2012	18	67.7	29.5	a	-	-
		13			b	Yes	NR
Grosso, M.J. [24]	2012	17	66.5	35.8	a	-	-
Romanò, C.L. [38]	2012	17	63	41.1	b	Yes	NR
Achermann, Y. [48]	2013	6	61	49	b	No	-
Boileau, P. [41]	2013	2	67	34	a	-	-
		4			b	No	-
Ghijselings, S. [49]	2013	3	65	56.3	b	Yes	NR
Klatte, T. O. [21]	2013	35	66	56.4	a	-	-
Magnan, B. [50]	2014	5	70.7	40.8	b	Yes	Gentamicin (0.8 g)/ Vancomycin (1 g)
Middernacht, B. [42]	2014	19	71	41.2	a	-	-
		4			b	No	-
Ortmaier, R. [51]	2014	12	65.2	24	b	Yes	NR
Black, E.M. [52]	2015	2	68.6	58.9	b	Yes	NR
Jacquot, A. [43]	2015	5	71	36	a	-	-
		14			b	Yes	NR
Morris, B.J. [53]	2015	6	60.6	38.1	b	No	-
Zhang, A.L. [54]	2015	11	69	24	b	Yes	Tobramycin (1.2 g)/ Vancomycin (1 g)
Hsu, J.E. [25]	2016	27	63.5	45.8	a	-	-
Piggott, D.A. [44]	2016	4	62	24	a	-	-
		7			b	Yes	NR
Stephens, B.C. [45]	2016	13	66.7	24	a	-	-
		5			b	Yes	NR
Assenmacher, A.T. [55]	2017	35	65	49.2	b	Yes	Gentamicin (2 g)/ Vancomycin (2 g)
Buchalter, D.B. [18]	2017	19	63	63	b	Yes	NR
Padegimas, E.M. [14]	2017	27	65,4	24	b	Yes	NR
Stone, G.P. [46]	2017	55	69.5	45	a	-	-
		19			b	Yes	NR
Grubhofer, F. [56]	2018	24	62	52	b	Yes	Gentamicin (0.55 g)/ Vancomycin (1 g)
Lee, S.H. [57]	2018	12	69.5	40.9	b	No	-
Merolla, G. [58]	2018	8	69.2	49	b	Yes	NR
Sevelda, F. [26]	2018	14	71	69.6	a	-	-
Torrens, C. [59]	2018	21	67.5	24	b	Yes	Tobramycin
Patrick, M. [60]	2019	27	67.8	12	b	Yes	Vancomycin (1 g)
Pellegrini, A. [61]	2019	11	66.6	96	b	Yes	Gentamicin/Vancomycin
Tseng, W.J. [62]	2019	27	66.4	32	b	Yes	Tobramycin (1.2 g)/ Vancomycin (1 g)
Boelch, S.P. [63]	2020	23	72	76	b	Yes	Gentamicin/Vancomycin
Brown, M. [64]	2020	25	70.2	38.3	b	Yes	Gentamicin/Clindamycin
Akgün, D. [65]	2021	35	67.1	61.2	b	Yes	NR
Bdeir, M. [15]	2021	19	66.1	57.6	b	Yes	Gentamicin/Vancomycin
Hornung, S. [66]	2021	13	68.2	13.2	b	Yes	NR
Klingebiel, S. [67]	2021	16	65	33.2	b	Yes	NR
Lemmens, L. [19]	2021	1	71	36	a	-	-
		23			b	Yes	NR
Meshram, P. [68]	2021	17	64	60	b	Yes	NR
Vilchez, H.H. [47]	2021	6	67.5	12	a	-	-
		15			b	No	-
Kim, D.H. [69]	2022	2	66	28	b	Yes	Vancomycin (4 g)
Schiffman, C.J. [27]	2022	35	55.5	164.5	a	-	-
Yao, J.J. [28]	2022	92	65.1	49.2	a	-	-

NR: not recorded.

**Table 2:** Overview of the organisms detected.

Organism	One-Stage (n)	Two-Stage (n)
*Pseudomonas aeruginosa*	2	4
*Alcaligenes*	2	0
*Cutibacterium acnes*	367	309
Coagulase-negative staphylococci (CNS)	80	20
*Corynebacterium*	5	4
*Staphylococcus aureus*	140	17
*Staphylococcus epidermidis*	89	21
*Staphylococcus capitis*	51	0
MRSA	2	7
MSSA	0	9
*Enterococci*	1	4
*Enterobacter cloacae*	0	1
*Enterococcus faecalis*	1	3
*Escherichia coli*	1	2
*Streptococcus Pneumoniae*	3	2
*Streptococcus oralis*	1	0
*Streptococcus dysgalactiae*	0	1
*Citrobacter freundii*	0	1
*Bacillus subtilis*	0	1
*Mycobacterium tuberculosis*	2	0
*Serratia*	0	1
*Klebsiella*	0	1
No growth	1	16

MRSA, methicillin-resistant Staphylococcus aureus; MSSA, methicillin-susceptible Staphylococcus aureus.

**Table 3:** Data of the outcome for both groups.

Parameter ± SD	Total	One-Stage	Two-Stage	*p*-Value
Age (Years)	65.76 ± 4.28	65.91 ± 4.67	65.61 ± 3.89	0.7896
Follow-up (Months)	45.67 ± 27.49	44.91 ± 31.79	38.74 ± 16.91	0.4425
CRP (mg/dL)	3.24 ± 2.91	3.7 ± 4.12	3.85 ± 3.07	0.9191
Revisions	1.84 ± 0.56	1.16 ± 0.18	2.25 ± 0.33	<0.0001 *
Abduction (°)	88.47 ± 22	103.38 ± 40.31	87.22 ± 20.61	0.4208
Elevation (°)	96.89 ± 19.10	101.47 ± 20.01	93.42 ± 17.20	0.5530
CS (Points)	47.25 ± 10.29	51.82 ± 9.17	45.22 ± 12.07	0.1523
ASES (Points)	63.39 ± 5.28	57.30 ± 3.72	67.06 ± 5.85	0.0326
SST (Points)	6.49 ± 5.28	5.34 ± 1.72	11.43 ± 18.50	0.3526
Reinfection rate (%)	12.3 ± 2.33	10.9 ± 2.77	12.9 ± 1.89	0.0062 *

SD, standard deviation; CS, constant score; ASES, American Shoulder and Elbow Surgeons Shoulder Score; SST, simple shoulder test; CRP, C-reactive protein, * statistically significant.

## Data Availability

The data presented in this study are available on request from the corresponding author.
